# Complex Adaptations Can Drive the Evolution of the Capacitor [*PSI* ^+^], Even with Realistic Rates of Yeast Sex

**DOI:** 10.1371/journal.pgen.1000517

**Published:** 2009-06-12

**Authors:** Cortland K. Griswold, Joanna Masel

**Affiliations:** Department of Ecology and Evolutionary Biology, University of Arizona, Tucson, Arizona, United States of America; University of Tennessee, United States of America

## Abstract

The [*PSI^+^*] prion may enhance evolvability by revealing previously cryptic genetic variation, but it is unclear whether such evolvability properties could be favored by natural selection. Sex inhibits the evolution of other putative evolvability mechanisms, such as mutator alleles. This paper explores whether sex also prevents natural selection from favoring modifier alleles that facilitate [*PSI^+^*] formation. Sex may permit the spread of “cheater” alleles that acquire the benefits of [*PSI^+^*] through mating without incurring the cost of producing [*PSI^+^*] at times when it is not adaptive. Using recent quantitative estimates of the frequency of sex in *Saccharomyces paradoxus*, we calculate that natural selection for evolvability can drive the evolution of the [*PSI^+^*] system, so long as yeast populations occasionally require complex adaptations involving synergistic epistasis between two loci. If adaptations are always simple and require substitution at only a single locus, then the [*PSI^+^*] system is not favored by natural selection. Obligate sex might inhibit the evolution of [*PSI^+^*]-like systems in other species.

## Author Summary

Can evolvability evolve? One obvious way to evolve faster is via mutator alleles that increase the mutation rate. Unfortunately, recombination will rapidly separate a mutator allele from the advantageous alleles that it creates. Mutators, therefore, gain very little benefit from promoting adaptations and are thought not to evolve in sexual organisms. Here we find that the [*PSI^+^*] prion, unlike mutator alleles, will evolve to promote evolvability in sexual yeast species. Together with previous laboratory studies of [*PSI^+^*]–mediated adaptation, and with bioinformatic studies consistent with [*PSI^+^*]–mediated adaptation in the wild, our theoretical results firmly establish [*PSI^+^*] as a model system for the evolution of evolvability. We also shed light on the importance of complex adaptations involving multiple genes. Adaptations involving multiple simultaneous genes drive the evolution of evolvability in this system. This work is an important proof of principle, showing that evolvability can sometimes evolve under realistic conditions.

## Introduction

[*PSI^+^*] is the aggregated prion form of the protein Sup35 [1]. [*PSI^+^*] aggregates appear spontaneously at a low rate [2]. Once established, [*PSI^+^*] causes normal Sup35 proteins to misfold to form more [*PSI^+^*] [3]. This self-catalytic conversion allows for transgenerational inheritance [4].

The normal, non-prion form of Sup35 is involved in stop codon recognition during gene translation [5],[6]. The depletion of normal Sup35 through its incorporation into prion aggregates leads to readthrough errors at stop codons [7]. This phenotypically reveals previously cryptic genetic variation beyond stop codons [8]–[11]. Revealed variation can sometimes lead to faster growth [10] and adaptation [12] under stressful lab conditions. These observations have controversially suggested a role for [*PSI^+^*] in promoting evolvability [13]–[17]. [*PSI^+^*] may tap into stocks of variation at times of stress when they are most likely to be needed [18].

**Figure 1:**
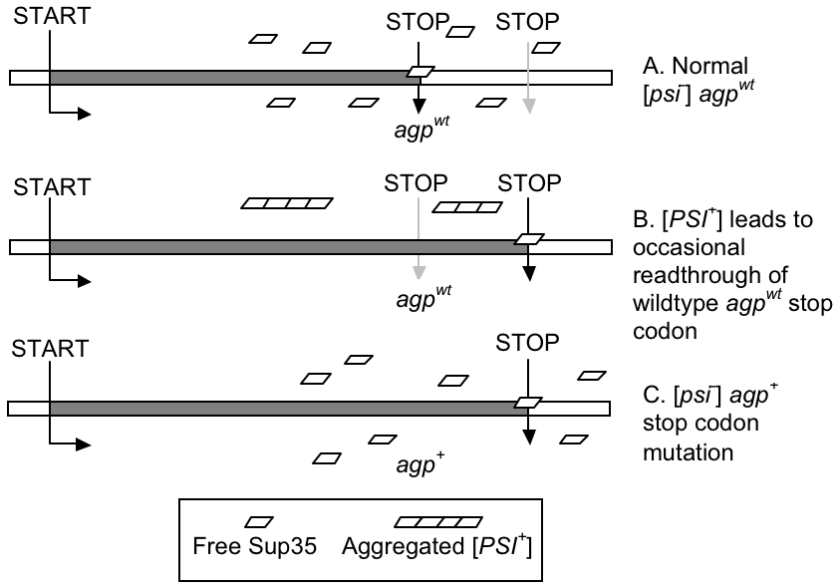
Different mechanisms of readthrough translation. Either the presence of [*PSI^+^*] (B) or an *agp* ^+^ point mutation (C) can lead to readthrough of the wild-type stop codon (A).

[*PSI^+^*] induces only low levels of any given adaptive readthrough product. A simple point mutation at the stop codon will produce much higher levels. Let the ancestral allele at locus *i* with an intact stop codon be designated 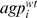 (*a*ppended *g*ene *p*roduct - *sensu* Masel and Bergman [15]) and the derived, adaptive allele with its stop codon destroyed by mutation be designated 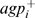 (Figure 1). [*PSI^+^*] promotes evolvability by acting as a stopgap mechanism. [*PSI^+^*] spontaneously appears far more often than stop codon mutations ([2],[19],[20]; see parameter estimates below). This provides partial but nevertheless rapid and therefore easily accessible adaptation. [*PSI^+^*] buys time for the lineage to expand, providing more opportunities for more precise adaptation later through genetic assimilation via the appearance of the stop codon mutant *agp* ^+^ [9],[14].

[*PSI^+^*] may provide a convenient model system for the more general study of evolvability via evolutionary capacitors [21]. Evolutionary capacitors are molecular mechanisms that act as switches to control the storage and release of cryptic genetic variation. Cryptic stocks of variation are likely to be pre-enriched for potential adaptations, making this mechanism of evolvability more potent than a reliance on new mutation [22].

Despite these experimental and theoretical results, a role for [*PSI^+^*] in evolvability has remained controversial. In particular, although data directly show that [*PSI^+^*] can sometimes promote rapid growth and adaptation in novel environments [10],[12], this does not imply that indirect selection for evolvability explains the emergence and evolutionary conservation of the [*PSI^+^*] system.

Theoretical results support the evolution of the evolvability properties of the [*PSI^+^*] system, but all such studies to date have neglected sex [15],[21],[23],[24]. This is of concern, since the evolution of another putative evolvability mechanism, namely mutator alleles, is dramatically inhibited by sex [25]–[27]. This is because recombination rapidly breaks up linkage between mutator alleles and the adaptations they generate, severely limiting the mutator's ability to hitchhike on the success of those adaptations. This argument does not, however, apply in an identical form to the [*PSI^+^*] system, since revealed variants remain dependent on continued [*PSI^+^*] expression, preventing their adaptive separation by recombination until genetic assimilation has occurred [15]. When linkage equilibrium evolves only slowly, evolvability may be favored by natural selection [28].

Here we examine for the first time the effect of realistic rates of Saccharomyces sex on the evolution of the evolvability properties of the [*PSI^+^*] system. Consider a modifier locus *prf* (*pr*ion-*f*orming - *sensu* Masel and Bergman [15]) that affects whether [*PSI^+^*] is formed. Examples of modifiers of [*PSI^+^*] formation in nature include the [*PIN* ^+^] prion [29], chaperone molecules [30]–[32] and changes in the Sup35 sequence [33],[34]. In our analysis, *prf* is an abstract modifier in the tradition of theoretical population genetics, rather than a specific, empirically identified modifier. Let the *prf^0^* allele completely suppress *de novo* [*PSI^+^*] formation and the *prf^+^* allele allow for it. We track allele frequencies at the *prf* locus in order to infer whether the [*PSI^+^*] system is favored by natural selection. Both alleles allow propagation of [*PSI^+^*], once present.

Usually, [*PSI^+^*] is deleterious, and so the *prf^+^* allele incurs small ongoing costs by generating [*PSI^+^*] lineages. But on rare occasions, [*PSI^+^*] and hence *prf^+^* may be adaptive. The *prf^0^* allele avoids the costs, but is still partially able to usurp the benefits by acquiring the cytoplasmically inherited [*PSI^+^*] element through sex with a [*PSI^+^*] strain. *prf^0^* can therefore be thought of as a “cheater” allele. When outcrossed sex is rare, however, as it is in Saccharomyces [35], *prf^0^* will on average acquire [*PSI^+^*] only after a potentially significant delay, during which a *prf^+^* lineage may have already hitchhiked to high frequency in association with [*PSI^+^*]-facilitated adaptation. Here we determine whether *prf^+^* is able to outcompete *prf^0^*, implying that the [*PSI^+^*] system is favored by natural selection on evolvability, given empirically estimated [35] rates of sex in Saccharomyces.

An interesting aspect of evolutionary capacitors in general, and the [*PSI^+^*] system in particular, is the fact that variants at many loci are exposed simultaneously. It has long been speculated that certain adaptations might involve multiple simultaneous changes, and that a temporary period of relaxed selection would allow multiple mutations to accumulate, providing greater diversity as the raw material for evolution [10],[36]. Of course, a potential problem with this idea is that cryptic genetic variation may also contain an accumulation of highly deleterious mutations. This may thwart adaptation, since revealing a stock of variation that includes both highly deleterious and mildly adaptive mutations will on balance likely be deleterious. However, capacitors such as [*PSI^+^*] tap into stocks of cryptic genetic variation that had remained subject to low levels of selection while in the cryptic state [22]. This low level of “pre-selection” is sufficient to weed out strongly deleterious alleles, while allowing mutations of small effect to accumulate [22]. One consequence of this pre-selection is that when variation is finally released through a capacitor, adaptations involving multiple simultaneous changes occur far more readily than they would without a capacitance mechanism [22].

Here we consider the evolution of the [*PSI^+^*] system via the *prf* modifier locus in the presence of sex, a fluctuating environment in which [*PSI^+^*] occasionally promotes adaptation, and both with and without complex adaptations involving multiple loci. We find that in the presence of realistic frequencies of Saccharomyces sex, complex adaptations are both necessary and sufficient for natural selection on evolvability to drive the evolution of the [*PSI^+^*] system.

## Materials and Methods

### Overview

The simulated diploid Saccharomyces population experiences a fluctuating environment. All environments where [*PSI^+^*] is deleterious we label “1” and the environments where [*PSI^+^*] generates an adaptation we label “2”. The probability of switching from environment 1 to 2 is Ω_12_ per generation, and the probability of switching from environment 2 to 1 is Ω_21_ per generation. We explore environmental switching rates between 10^−7^ and 10^−3^ per generation. The population starts in environment 1, with *prf^+^* and *prf^0^* allele frequencies of 0.5, and evolves for 5/Ω_12_ generations. This process is replicated to determine the proportion of runs for which the *prf^+^* frequency increases. Model parameters are listed in Table 1, including default values when not otherwise specified.

**Table 1:** Parameters in the Model.

Parameter	Definition	Value and supporting references
*δ_psi−_*	Probability of stop codon readthrough in [*psi^−^*] individuals	0.003 [7]
*δ_PSI+_*	Probability of stop codon readthrough in [*PSI^+^*] individuals	0.01 [7]
*μ*	Per stop codon mutation rate (forwards and backwards) per replication	1.3×10^−9^ [19]
*m*	Probability of [*PSI^+^*] formation per diploid offspring	10^−5^ [2, Tuite MF, pers comm]
*m′*	Probability of [*PSI^+^*] loss per diploid cell division	10^−5^ [38]
*p_sex_*	Probability an offspring is formed sexually	0.001 [35], also 0.01, 0.1
*p_auto_*	Given sex, probability of automixis (within tetrad mating)	0.94 [35]
*p_amphi_*	Given sex, probability of amphimixis (random mating)	0.01 [35]
*p_haplo_*	Given sex, probability of haplo-selfing (mother-daughter mating)	0.05 [35]
*N_e_*	Effective population size	5×10^6^ [19],[20],[35]
*ε*	Frequency of [*PSI* ^+^] in wild *prf^+^* populations, used to infer selection against [*PSI* ^+^] in environment 1	0.01 and lower [39],[40]
*α*, *s*_1_	parameters inferred, primarily from *ε* and *m*, that determine the strength of selection against [*PSI* ^+^] in environment 1	
*s*_2_	Strength of selection for [*PSI* ^+^]Agp^wt^ in environment 2	0.1-0.001
*h*	Dominance of *prf^+^*	{0,1}
Ω_12_	Per generation switching probability from environment 1 to 2	10^−7^–10^−5^
Ω_21_	Per generation switching probability from environment 2 to 1	10^−5^–10^−3^
*k_−_*/*k_+_*	Equilibrium constant of adaptive dimerization in expression concentration units (see Text S2)	10^−3^
*k_1_*	Rate constant associated with adaptive readthrough dimer function, per unit concentration (see Text S2)	50

In environment 2, [*PSI^+^*] can mediate adaptation by expressing novel gene product(s) at either one locus (simple adaptation; *i* = 1) or two loci (complex adaptation; *i*∈{1,2}). Our “*agp*” notation and our parameter estimates are based on the assumption that adaptation comes from addition to a protein C-terminal through stop codon readthrough. The same formalism can, however, still be applied if the variation revealed by [*PSI^+^*] is instead mediated via nonstop mRNA decay [11], via +1 frameshifting at shifty-stop sites [37], or via variation in genes regulated downstream through any of these mechanisms [37]. Each switch to environment 2 is considered unique and involves a new set of *agp* loci, whose frequencies are initialized at this time. After switching back to environment 1, this set of *agp* loci is no longer tracked.

**Figure 2:**
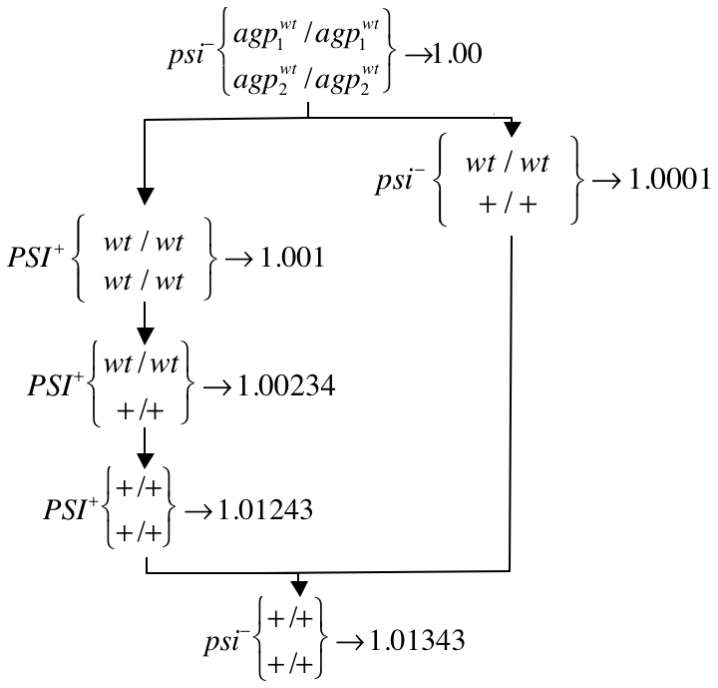
Alternative pathways, with and without [*PSI^+^*], leading to the same readthrough adaptation. Adaptation in environment 2 can proceed either via: (1) [*PSI^+^*] appearance followed by point mutation at *agp* loci (genetic assimilation) and finally reversion to [*psi* ^−^] (left) and (2) direct adaptation at *agp* loci without involvement of [*PSI^+^*] (right). The fitness of an individual is given to the right of its genotype, calculated using *s*_2_ = 0.001. Only homozygous states are shown because inbreeding quickly leads to homozygosity. The genetic assimilation pathway typically occurs more often because [*PSI^+^*] individuals appear far more often than [*psi^−^*] individuals who carry the “+” allele at both *agp* loci.

If only a single *agp* ^+^ allele is required for adaptation, and it is already present or very soon appears in the population, then adaptation will proceed via this more direct route rather than via [*PSI^+^*], yielding no benefit to a *prf^+^* allele. If, however, two different readthrough products are simultaneously involved in a complex adaptation, then it becomes exceedingly unlikely that both 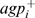 alleles will initially be present in the same individual. In this case [*PSI^+^*] will have an advantage, since it will cause simultaneous readthrough at both loci, reaping synergistic benefits and promoting complex adaptations. Competing paths of direct vs. [*PSI^+^*] mediated adaptation are shown in Figure 2 for the two locus case.

### Individual Genetic and Cytoplasmic States

We track individual genotypes at the *agp* and *prf* loci. Haploids must have different alleles (α vs. *a*) at the mating-type *mat* locus in order to conjugate, and so we also track the *mat* locus for its potential effect on inbreeding. We do not model mutation at the *mat* and *prf* loci, except implicitly through the possibility of mother-daughter haplo-selfing (see Reproduction below). There is free recombination between all loci. Individuals therefore have either three or four genetic loci, depending on whether we are modeling simple or complex adaptations with one or two *agp* loci, respectively. Each of the three to four loci has two alleles, plus there are also two possible cytoplasmic states ([*PSI^+^*] versus [*psi^−^*]).

### Mutation Rate at *agp* Locus

The point mutation rate in Saccharomyces is around 5×10^−10^ per base pair per cell division [19]. We approximate the frequencies of the 3 stop codons TAA, TAG and TGA as equal and all mutational substitution types as equally likely. All point mutations at the first position destroy the stop codon. So do all but two at the second position (namely those between TAA and TGA) out of 3 possible substitutions at each of the 3 stop codons. Similarly, of the 9 possible substitutions at the third position, only those between TAA and TAG preserve the stop codon. The total rate of stop codon destruction by point mutation is therefore estimated as (1+7/9+7/9)×5×10^−10^ = 1.3×10^−9^ per cell division.

Although mutations that precisely reverse stop codon loss are rarer than this, compensatory mutations can also create alternative stop codons nearby, leading to a functionally equivalent gene product. We therefore assume symmetric mutation rates at the *agp* loci. The back mutation rate is primarily important only for setting *agp* allele frequencies at mutation-selection-drift equilibrium in environment 1 (see Text S1).

### [*PSI^+^*] Appearance and Loss

We explore both the case where the *prf^+^* allele is completely dominant (*h* = 1), and the case where it is completely recessive (*h* = 0) relative to the *prf^0^* allele. *prf^+^* individuals form [*PSI^+^*] with probability *m* = 10^−5^ per generation [2, Tuite MF pers. comm.]. [*PSI^+^*] is lost with probability *m′* per generation during cell division. Empirical work shows that *m′*<0.0002 [38]; here we follow the common assumption that *m′*≈*m*.

Note that *m* increases by as much as 60-fold in response to environmental stress [18]. This responsiveness increases the ability of *prf^+^* to promote evolvability. Here we make the conservative assumption that *m* does not depend on the degree of adaptation to the current environment.

### Fitness

In both environments, readthrough products at any locus induced either by [*PSI^+^*] or by point mutations in stop codons are likely to incur a fitness cost. This cost could be related to gain or loss of function, and hence specific to the gene in question, or it could be a more general metabolic cost. Here, in order to develop a general, parameterized model, we assume a metabolic cost.

In environment 2, the metabolic cost of readthrough is ameliorated because of the adaptive effects of a substrate-dimer reaction involving readthrough products at one or two loci. Readthrough probabilities are *δ_psi−_* = 0.003 and *δ_PSI+_* = 0.01 in [*psi^−^*] and [*PSI^+^*] cells respectively [7]. Let *E_i_* be the level of readthrough at locus *i*. *E_i_* is equal to *δ_j_* for 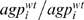 genotypes, (1+*δ_psi−_*)/2 for 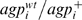 genotypes and 1 for 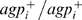 genotypes, where *j*∈{*psi*−, *PSI*+}. The unit concentrations for all equations below is now given relative to a typical expression level of a gene defined as *E* = 1.

The fitness of an individual in environment 1 is
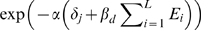
(1)where *L* is either 1 or 2, depending on whether simple or complex adaptation is assumed, and *β_d_* is a constant that weights the metabolic cost of readthrough at the potentially adaptive loci relative to the metabolic cost of readthrough across the whole genome. Since there are ∼5000 genes in Saccharomyces, we assume that *β_d_* = 1/5000. Equation 1 yields a fitness of 1 in the absence of readthrough, decaying exponentially towards zero as levels of readthrough increase. The parameter *α* controls the strength of selection against readthrough.

In environment 2, an individual's fitness depends both on the metabolic cost above, and on a benefit accruing from readthrough at *agp* loci. We assume that the readthrough *Agp^+^* gene product has adaptive function when in the form of a dimer. For simple adaptations, this is an *Agp^+^* homodimer. For complex adaptations, this is an 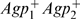 heterodimer. These dimeric scenarios allow us to capture synergistic epistasis in a realistic way that allows direct comparison between one-locus and two-locus models. Fitness in environment 2 is given by
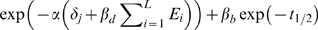
(2)where *β_b_* is a parameter controlling the magnitude of the adaptive effects and *t*_1/2_ is the half-life of a substrate acted on by a catalytic *Agp^+^* dimer. The first term represents the metabolic cost of readthrough, and is identical to fitness in environment 1. *β_b_* is set such that the relative fitness of [*PSI^+^*] homozygous *agp* ^+^ individuals is 1+*s*_2_ where *s*_2_ = 0.001, 0.01 or 0.1. Fixing *s*_2_ in this way allows appropriate comparisons between the 1-locus and 2-locus models. *t*_1/2_ captures how the strength of adaptation depends on the extent of readthrough at each of the *L* loci. The biochemical model for calculating *t*_1/2_ depends on the *Agp^+^* dimer concentration and is presented in the Text S2 and Figure S1.

Masel and Griswold [39] estimate the strength of selection against [*PSI^+^*]. This estimate depends on the frequency of [*PSI^+^*] as a rare polymorphism in wild, [*PSI^+^*]-competent Saccharomyces populations. Following expression of a Sup35-GFP fusion protein, a few cells from wild populations show aggregates almost immediately [40]. This suggests the pre-existence of [*PSI^+^*] cells containing Sup35 aggregates at a frequency of *ε* = 1%. If some of these aggregates are false positives, then the true value of *ε* could be lower. Assuming populations are in epimutation-selection balance, the strength of selection against [*PSI^+^*] is [39]

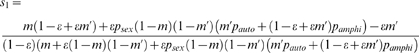
(3)where *p_sex_* is the probability an offspring is formed sexually. Given that an individual is formed sexually, *p_auto_* is the probability it is formed via automixis and *p_amphi_* is the probability it is formed via amphimixis (see below). Although Equation 3 is complex, its inference of the strength of selection against [*PSI^+^*] depends largely just on the observed [*PSI^+^*] frequency *ε* and the rate of [*PSI^+^*] appearance *m* [39].

Given selection *s_1_* against [*PSI^+^*] in environment 1, *α* is given by
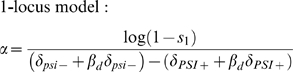
(4a)

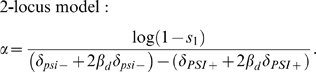
(4b)Equation 4 is derived from Equation 1 by equating 1−*s*_1_ to the fitness of [*PSI^+^*] individuals relative to *psi^−^* individuals. We use*ε* to calculate *s*_1_ and hence *α*, and *α* to run our simulations. Since there is uncertainty in the estimate of the equilibrium frequency *ε* of [*PSI^+^*] when deleterious, we explore cases when *ε* is 0.01%, 0.1% and 1%.

### Simulated Evolution

We analyze evolutionary competition between *prf^+^* and *prf^0^* alleles by initializing a population in environment 1 with a 0.5 frequency of each allele, and simulating evolution for 5/Ω_12_ generations to determine how often *prf^+^* increases in frequency.

We use mutation-selection-drift balance theory to initialize [*PSI^+^*] frequencies, and also to initialize *agp* frequencies at the moment when the switch to environment 2 occurs (see Text S1 for details). We assume initial linkage equilibrium between all loci. Although epimutation tends to associate [*PSI^+^*] with *prf^+^*, we neglect this association during initialization since it is not tractable, and since in any case it establishes itself very rapidly on the timescale of our simulations. Reduced covariance between *prf^+^* and [*PSI^+^*] prior to a change from environment 1 to 2 inhibits the maintenance of *prf^+^* (pers. obs.) and so the approximation of linkage equilibrium is conservative relative to inferring whether *prf^+^* can be maintained.

Given genotype and epigenotype frequencies in one generation, we calculate the effects first of reproduction and epimutation (described below), then of mutation at the *agp* locus, and finally of selection (according to fitnesses described above) to calculate expected genotype frequencies in the next generation. We then sample realized genotype frequencies from expected genotype frequencies using the multinomial distribution to capture genetic drift in a finite population of size *N_e_*. The effective population size *N_e_* in Saccharomyces can be estimated as *θ*(1+*F*)/(4*μ*) where *θ* is the pairwise sequence divergence estimated as 0.0032–0.0038 [35], the inbreeding coefficient *F* = 0.98 [35], and the per-base pair per replication point mutation rate *μ* is around 3.3×10^−10^ [20] to 5×10^−10^ [19]. This yields *N_e_*≈3×10^6^–6×10^6^. We use *N_e_* = 5×10^6^.

### Reproduction and Epimutation

Saccharomyces is generally diploid, and normally reproduces asexually, with only around *p_sex_* = 0.1% of offspring formed via sex [35]. We ignore the haploid stage of the life cycle in our calculations of both mutation and selection, thus assuming that there is no fitness cost to sex in terms of a delay in forming the next generation of diploid offspring. We calculate a combination of sexual and asexual diploid offspring produced instantaneously in each generation.

Given sex, only around *p_amphi_* = 1% of offspring are generated through amphimictic random mating in the population [35]. *p_auto_* = 94% of sexual offspring are formed by automictic within-tetrad mating, while *p_haplo_* = 5% of sexual offspring are formed when the products of a haploid mother-daughter mitotic division mate with one another following mating-type switching [35]. In our simulations we explore the effect of varying the overall probability of sex *p_sex_*, but hold the relative proportions of amphimixis *p_amphi_*, automixis *p_auto_* and haplo-selfing *p_haplo_* constant at the values estimated by Tsai et al. [35]. Amphimictic and automictic mating are only allowed to occur between cells of opposite mating type specified at the *mat* locus. All sexual reproduction involves independent segregation at each genetic locus.

Propagation of [*PSI^+^*] state is slightly more complex. Both sexual and asexual reproduction consist of cell division followed by cell growth. During cell division, [*PSI^+^*] is lost with probability *m′*. During subsequent cell growth, [*PSI^+^*] appears spontaneously in *prf^+^* cells with probability *m*. When reproduction is sexual, both contributing individuals first have the opportunity to lose [*PSI^+^*] during meiosis with probability *m′*. The new diploid individual is then [*psi^−^*] only if both parent cells are [*psi^−^*]. This allows *prf^0^* lineages to capture the benefits of [*PSI^+^*]. During diploid cell growth following mating, [*PSI^+^*] has a single opportunity to appear with probability *m* in *prf^+^* cells.

### Simulation from Initially Rare *prf^+^* Mutants

Some simulations were initialized with only a single *prf^+^* mutant rather than with a 50% allele frequency. This single mutant appears in a random genetic background, and in environment 1 rather than environment 2 with probability Ω_21_/(Ω_12_+Ω_21_). When *prf^+^* appeared in environment 1, simulations were carried out in the same way as for an initial 0.5 frequency described above. For single mutants, simulations continued forward in time until *prf^+^* went either extinct or fixed in the population, rather than observing whether its frequency was greater or less than 0.5 after a certain number of generations. Fixation probability was then compared to the neutral expectation of 1/*N*.

When *prf^+^* appeared in environment 2, simulations began at the time of the previous environmental switch from 1 to 2. Both the time *t_prf+_* of the appearance of the *prf^+^* allele by mutation and the time *t_21_* of switching back to environment 1 were preset as follows. First, *t_prf+_* and *t_21_* were drawn from geometric distributions with mean 1/*μ_prf+_* and 1/Ω_21_ respectively where *μ_prf+_* is the probability a *prf^+^* allele arises per generation and was set to an arbitrarily low value. Then while *t_prf+_*>*t_21_*, we reset *t_21_* to equal *t_21_*−*t_prf+_*. It is important to note that it is possible for the population to adapt to environment 2 prior to the arrival of the *prf^+^* allele. If the population adapts prior to the arrival of the *prf^+^* allele, *prf^+^* will be unconditionally deleterious.

### Confidence Intervals

95% confidence intervals in the figures are calculated using the approximate method suggested by Agresti and Coull [41].

## Results

**Figure 3:**
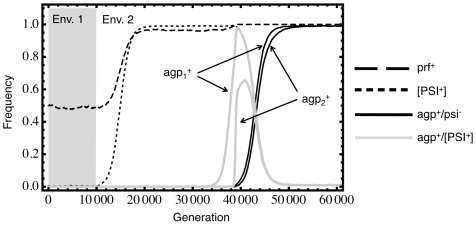
An example of two-locus adaptation mediated by [*PSI^+^*]. *ε* = 0.01, Ω_12_ = Ω_21_ = 10^−5^, *s*_2_ = 0.001, *h* = 1.

The simulated yeast population experiences a fluctuating environment between times when [*PSI^+^*] is deleterious and times when it is adaptive. A sample run showing *prf^+^* fixation is shown for illustrative purposes in Figure 3. *prf^+^* very slowly declines in frequency until the switch from environment 1 to 2 occurs. A [*PSI^+^*] selective sweep then immediately begins, with *prf^+^* hitchhiking to high frequency. Later, first one and then both rare *agp^+^* mutants appear. Once recombination has combined the two, a [*psi^−^*] revertent sweeps through the population. By this stage *prf^+^* has already become fixed.

### *prf^+^* Is Expected to Be Maintained in Yeast Even with Sex, Provided Adaptation Is Complex

**Figure 4:**
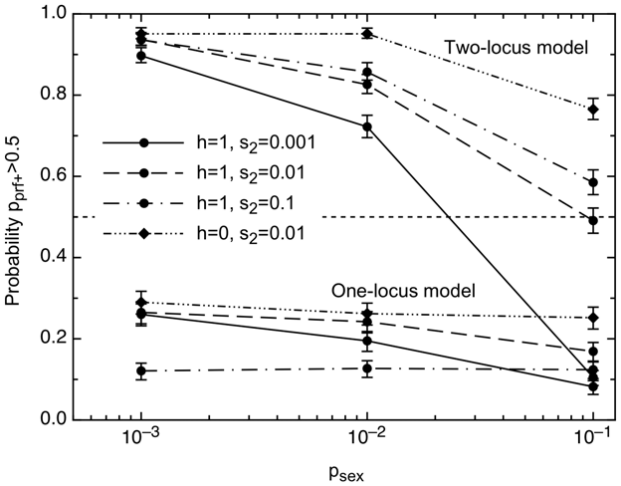
*prf^+^* is maintained in the two-locus but not the one-locus model. The *y*-axis gives the probability that the frequency of *prf^+^* after 5×10^5^ generations is greater than its starting frequency of 0.5. The strength of selection *s_2_* for adaptation in environment 2 affects the cutoff frequency of sex. ε = 0.01, Ω_12_ = Ω_21_ = 10^−5^. *prf^+^* is maintained in the two-locus model unless sex is very frequent.

We see in Figure 4 that with *p_sex_* = 10^−3^, as estimated for *S. paradoxus* [35], *prf^+^* is favored given complex but not simple adaptations. This inference does not depend on the extreme rarity of yeast sex: with complex adaptations, *prf^+^* would still be maintained even if the probability of sex were raised an order of magnitude. Once sex becomes as frequent as 0.1, *prf^+^* is maintained only if selection on [*PSI^+^*]-mediated adaptations is strong. From these results, it seems unlikely that a [*PSI^+^*]-like evolvability system could be favored by natural selection in an obligately sexual species under the conditions considered here.

For most of our simulations, we assume *prf^+^* is dominant (*h* = 1). When *prf^+^* is completely recessive (*h* = 0), sex provides even less of a barrier to the evolution of evolvability (Figure 4).

### Uncertainty in the Strength of Selection against [*PSI^+^*]

Inference of the strength of selection against [*PSI^+^*] in Equation 3 depends on the estimate *ε* = 1% of the mean frequency of [*PSI^+^*] in *prf^+^* populations at mutation-selection-drift equilibrium (see [39] for details). This estimate may contain false positives and instead be an upper bound. In Figure S2, we see that uncertainty in *ε* is not important, since lower values of *ε*, implying stronger selection against [*PSI^+^*] in environment 1, lead us to the same conclusions.

### Environmental Switching Rates Ω_12_ and Ω_21_

**Figure 5:**
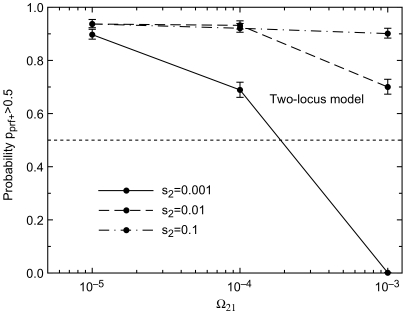
If environment 2 is too short-lived (high Ω_21_), then *prf^+^* is not maintained. This is because there is insufficient time for the selective sweeps shown in Figure 3 to be completed. ε = 0.01, Ω_12_ = 10^−5^, *h* = 1.

If environment 2 is too short-lived for selective sweeps to be completed, then capacitance cannot evolve (Figure 5). This agrees with previous work using a different modeling approach [42].

**Figure 6:**
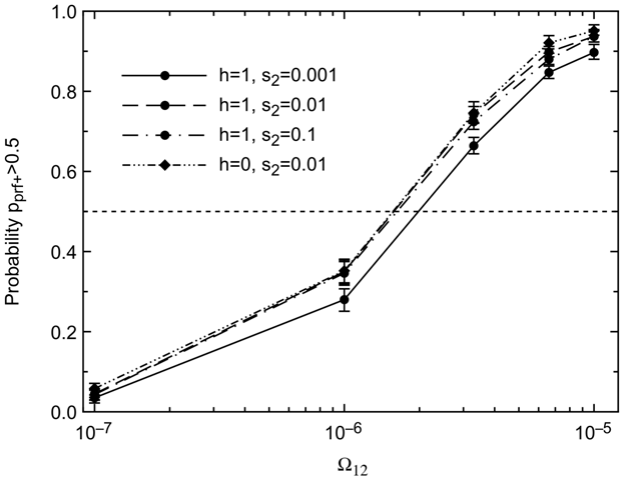
Very rare opportunities for adaptation (low Ω_12_) cause *prf^+^* to be lost. ε = 0.01, Ω_21_ = 10^−5^.

Opportunities for adaptation must also arise at a minimum frequency for capacitance to evolve (Figure 6). Previous work in an asexual model found that a capacitor must be useful at a minimum frequency of Ω_12_>1/*N_e_* per generation in order to be favored by natural selection [23]. With realistic levels of Saccharomyces sex (i.e., *p_sex_* = 0.001), we see in Figure 6 that *prf^+^* increases in frequency when Ω_12_>2×10^−6^, corresponding instead to Ω_12_ *N_e_*>10. This still corresponds to an exceptionally mild and plausible absolute requirement on the rate of environmental change.

### Fixation of Initially Rare *prf* ^+^ Mutants

**Figure 7:**
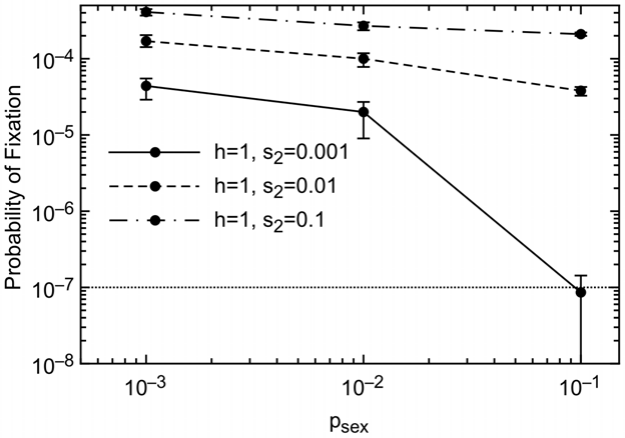
Fixation probabilities starting from a single copy. *prf^+^* fixes more often than the neutral expectation (10^−7^), except when selection is weak (*s*_2_≤0.001) and sex is common *p_sex_*≥0.1. All parameters are equal to values in Figure 3, with the additional parameter *μ_prf+_* equal to 10^−9^. For *p_sex_* = 0.001 and *p_sex_* = 0.01, results are based on 10^6^ replicates. For *p_sex_* = 0.1 and *s_2_* = 0.001, the number of replicates is 3.5×10^7^. Otherwise the number of replicates is 6×10^6^.

**Figure 8:**
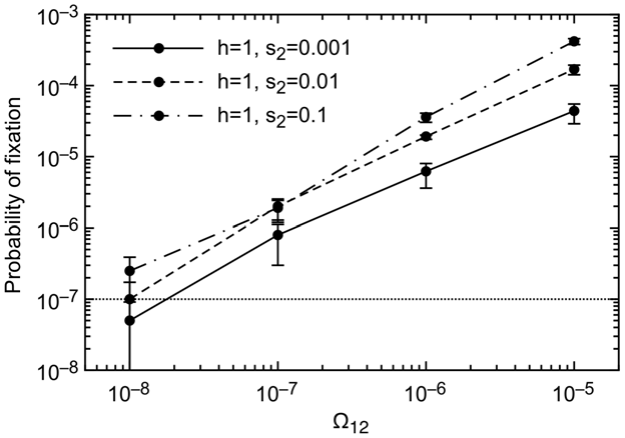
Fixation probabilities starting from a single copy. *prf^+^* allele fixes above neutral expectations (10^−7^) when the transition probability to environment 2 is greater than 10^−8^. All parameters are equal to values in Figure 6, with the additional parameter *μ_prf+_* = 10^−9^. Results are based on 10^6^ replicates (Ω_12_ = 10^−5^), 5×10^6^ replicates (Ω_12_ = 10^−6^), 2×10^7^ replicates (Ω_12_ = 10^−7^) and 2×10^7^ replicates (Ω_12_ = 10^−8^).

A *prf^+^* frequency of 0.5 is a very artificial starting condition, and was chosen for computational efficiency. To test the sensitivity of our results to this starting condition, we also did an invasion analysis starting with a single new *prf^+^* mutant (Figures 7 and 8). The neutral expectation of fixation with probability 1/*N* is shown by a dashed line. In agreement with results using a 0.5 starting condition (Figure 3), we find that *prf^+^* will fix with a probability greater than the neutral expectation, provided that sex is not too common and selection is not too weak (Figure 7). *prf^+^* fixes more often than the neutral expectation when Ω_12_≥10^−7^ (Figure 8), favoring evolvability at even lower levels of Ω_12_ than with a 0.5 starting condition (Figure 6), in agreement with previous work in an asexual model that Ω_12_>1/*N_e_* per generation is the necessary and sufficient condition for *prf^+^* to be favored by natural selection [23]. Our more comprehensive calculations above that began with a *prf^+^* frequency of 0.5 seem to be mildly conservative with respect to the evolution of evolvability.

## Discussion

When realistic levels of yeast sex are accounted for, indirect selection for evolvability can still favor the evolution of the [*PSI^+^*] system. This is only true, however, if adaptation involves at least two loci with synergistic epistatic effects on fitness. Otherwise, with an effective population size as large as that of yeast, all single-locus mutants are readily accessible through mutation. [*PSI^+^*] is a stopgap adaptation that incurs costs as well as benefits, and is never preferred to direct adaptation. However, simultaneous direct adaptation at multiple loci is extremely rare, and modifiers of [*PSI^+^*] hitchhike to high frequency by virtue of facilitating it.

Evolutionary capacitors, by exposing multiple variants simultaneously, have long been believed to facilitate complex adaptations involving multiple sites [10],[22],[36]. Here we find that the converse is also true: complex adaptations facilitate the evolution of capacitors. This illustrates the intricate relationship between the two.

Sex strongly inhibits the evolution of mutator genes, but here we find that its effect on modifiers of capacitance is much weaker. Nevertheless, were yeast to undergo obligate sex, this would be sufficient to disrupt the evolution of [*PSI^+^*] under a model of 2-locus adaptation. Our model is specific to the parameters of the [*PSI^+^*] system in Saccharomyces, and the evolution of other putative capacitors in the presence of sex still remains to be determined.

## Supporting Information

Figure S1Fitness contours in environment two as a function of 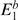 and 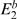. The approximation 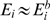 was used to calculate optimal expression. This approximation is reasonably accurate because *δ_j_*, *j*∈{*psi^−^,*[*PSI^+^*]} is small 0<*δ_j_*≤0.01.(4.09 MB TIF)

Figure S2 Values of ε lower than the upper bound ε = 0.01 do not change our results. Lower values of ε imply stronger selection against [*PSI^+^*] in environment one. This could inhibit [*PSI^+^*]-mediated adaptation, but the effect is negligible unless selection for [*PSI^+^*] in environment two is very weak. Ω_12_ = Ω_21_ = 10^−5^, *h* = 1.
(8.80 MB TIF)

Text S1 Initial allele frequencies.
(0.03 MB PDF)

Text S2 Adaptive gene product activity.
(0.03 MB PDF)
