# Supplementary figures and images for: Complex Adaptations Can Drive the Evolution of the Capacitor [PSI +], Even with Realistic Rates of Yeast Sex
Source: PLoS Genet. 2009 Jun 12;5(6):e1000517. doi: 10.1371/journal.pgen.1000517 (PMC2686163; doi:10.1371/journal.pgen.1000517)

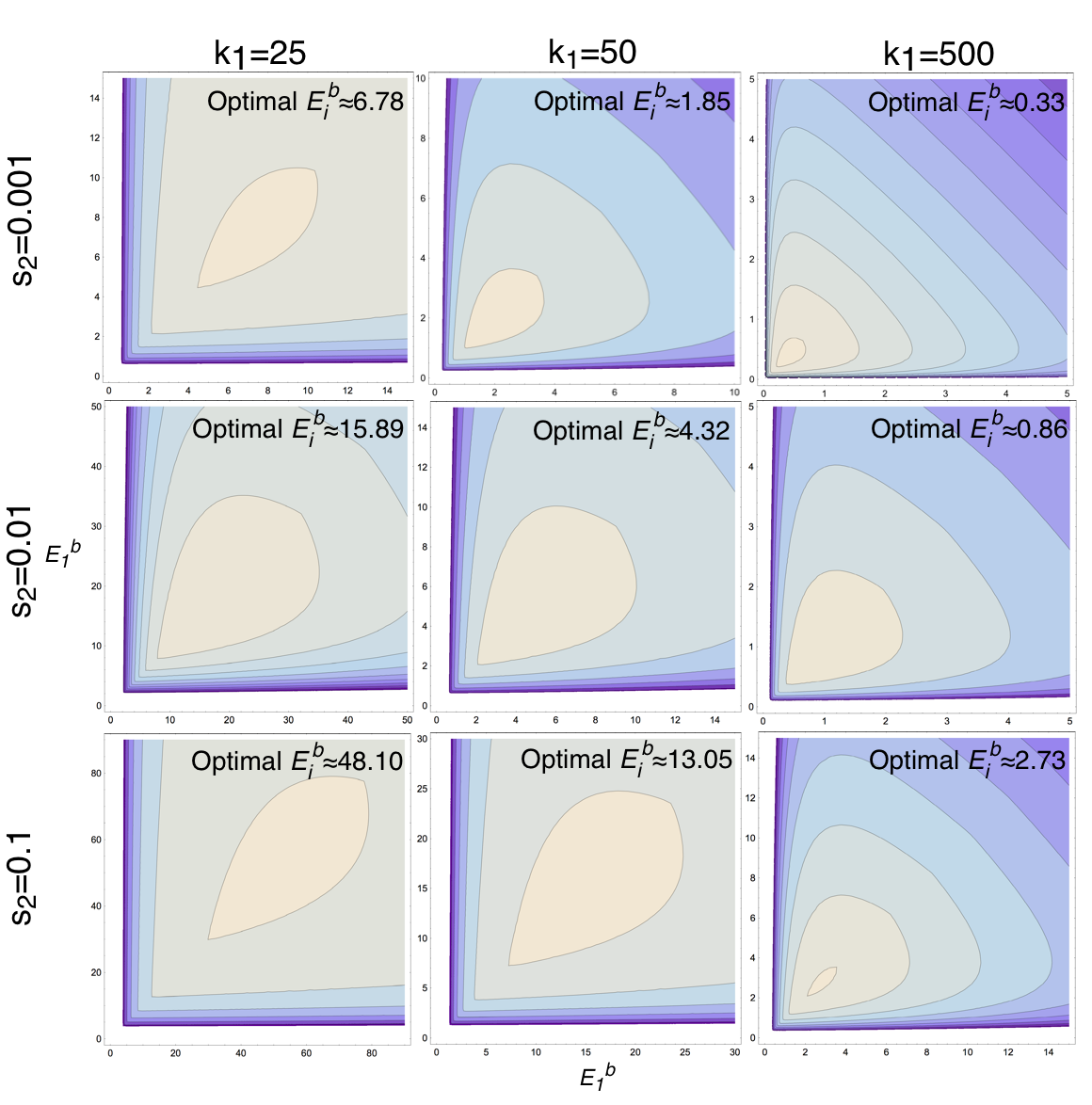

Supplement: Figure S1 — Fitness contours in environment two as a function of and . The approximation was used to calculate optimal expression. This approximation is reasonably accurate because δj, j∈{psi−,[PSI+]} is small 0<δj≤0.01. (4.09 MB TIF) [file pgen.1000517.s001.tif]

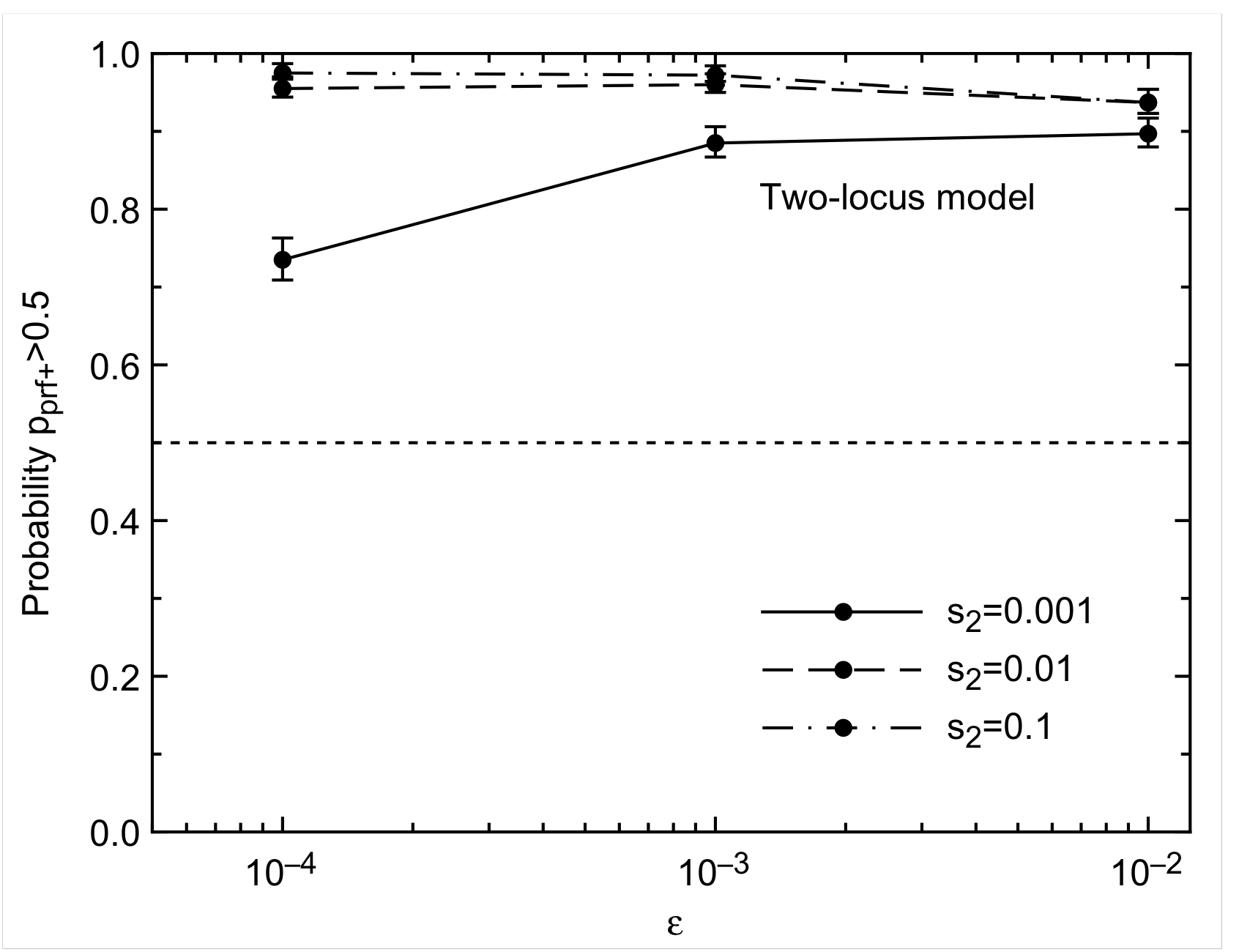

Supplement: Figure S2 — Values of ε lower than the upper bound ε = 0.01 do not change our results. Lower values of ε imply stronger selection against [PSI+] in environment one. This could inhibit [PSI+]-mediated adaptation, but the effect is negligible unless selection for [PSI+] in environment two is very weak. Ω12 = Ω21 = 10−5, h = 1. (8.80 MB TIF) [file pgen.1000517.s002.tif]
