# Supplementary material for: Complex Adaptations Can Drive the Evolution of the Capacitor [PSI +], Even with Realistic Rates of Yeast Sex
Source: PLoS Genet. 2009 Jun 12;5(6):e1000517. doi: 10.1371/journal.pgen.1000517 (PMC2686163; doi:10.1371/journal.pgen.1000517)
Supplement: Text S1 — Initial allele frequencies. (0.03 MB PDF) [file pgen.1000517.s003.pdf]

S1: Initial allele frequencies

We use mutation-selection-drift balance theory to initialize [PSI^+] frequencies, and also to initialize *agp* frequencies at the moment when the switch to environment two occurs. The frequency *x* of agp_i^+ $\left(i \in \{1,2\}\right)$ alleles at mutation-selection-drift balance follows Wright's distribution [1,2]:

$$\phi_{agp_i^+}(x) = C_{agp_i^+} x^{4N_e\mu-1}(1-x)^{4N_e\mu-1}\overline{w}_{agp_i}^{2N_e} \qquad \text{(S1.1)}$$

where $C_{agp_i^+}$ is a constant of integration such that $\int_0^1 \phi_{agp_i^+}(x)dx = 1$ and μ is the forward and reverse mutation rate estimated as 1.3 ×10^{-9}. $\overline{w}_{agp_i}$ is the average fitness at the agp_i locus in environment 1, which is

$$\begin{aligned} \overline{w}_{agp_i} &= \left(x^2 + x(1-x)F\right)w_{agp_i^+/agp_i^+} \\ &+ 2x(1-x)(1-F)w_{agp_i^+/agp_i^{wt}} + \left((1-x)^2 + x(1-x)F\right)w_{agp_i^{wt}/agp_i^{wt}}. \end{aligned} \qquad \text{(S1.2)}$$

where F=0.98 for Saccharomyces [3].

The equilibrium probability distribution of [PSI^+] in environment one can be derived in a similar way using a diffusion approach. Let *z* be the frequency of individuals with [PSI^+]. Then

$$E(\Delta z) = -mz + my(1-z) + \frac{z(1-z)}{\overline{w}}\frac{d\overline{w}}{dz} \qquad \text{(S1.3)}$$

where *y* is the frequency of the prf^+ allele and it is assumed that there is a high rate of inbreeding so that spread of [PSI^+] via outcrossing can be ignored for this calculation. $\overline{w}$ is the average fitness of an individual. The variance in the rate of change in frequency of [PSI^+] is $V(\Delta z) = z(1-z)/N_e$ assuming binomial sampling. The equilibrium distribution of [PSI^+] in environment one $\left(\phi_{PSI^+}(z)\right)$ is then [1]

$$\begin{aligned} \phi_{PSI^+}(z) &= \frac{C}{V_{\Delta z}}\exp\left(2\int E(\Delta z)/V(\Delta z)dz\right) \\ &= C(1-z)^{2N_e m-1} z^{2N_e my-1}\overline{w}^{2N_e} \end{aligned} \qquad \text{(S1.4)}$$

where *C* is a constant such that $\int_0^1 \phi_{PSI^+}(z)dz = 1$ and N_e is the effective population size.

References

1. Wright S (1938) The distribution of gene frequencies under irreversible mutation. Proc Natl Acad Sci USA 24: 253-259.
2. Wright S (1937) The distribution of gene frequencies in populations. Proc Natl Acad Sci USA 23: 307-320.
3. Tsai IJ, Bensasson D, Burt A, Koufopanou V (2008) Population genomics of the wild yeast *Saccharomyces paradoxus*: Quantifying the life cycle. Proc Natl Acad Sci USA 105: 4957-4962.
