# Supplementary material for: Complex Adaptations Can Drive the Evolution of the Capacitor [PSI +], Even with Realistic Rates of Yeast Sex
Source: PLoS Genet. 2009 Jun 12;5(6):e1000517. doi: 10.1371/journal.pgen.1000517 (PMC2686163; doi:10.1371/journal.pgen.1000517)
Supplement: Text S2 — Adaptive gene product activity. (0.03 MB PDF) [file pgen.1000517.s004.pdf]

S2: Half-life model of adaptive gene product activity

Assume that the adaptive gene product requires readthrough at the primary stop codon, but not at any subsequent "backup" stop codons in the 3'UTR. The relevant adaptive expression levels $\left(E_i^b\right)$ of the Agp_i^+ products are now $\delta_j(1-\delta_j)$ for agp_i^{wt}/agp_i^{wt} genotypes, $(1-\delta_j^2)/2$ for agp_i^{wt}/agp_i^+ genotypes and $(1-\delta_j)$ for agp_i^+/agp_i^+ genotypes for $i \in \{1,2\}$ and $j \in \{psi^-, PSI^+\}$. Let F_1^b and F_2^b give the portions of total gene product levels E_1^b and E_2^b that are free rather than contained in a dimer. Let k_+ and k_- be the rate constants of dimer assembly and disassembly respectively. Heterodimer formation is now described by

$$\frac{dF_1^b}{dt} = k_-[\text{dimer}] - k_+F_1^bF_2^b$$
$$\frac{dF_2^b}{dt} = k_-[\text{dimer}] - k_+F_1^bF_2^b$$
$$\frac{d[\text{dimer}]}{dt} = k_+F_1^bF_2^b - k_-[\text{dimer}].$$

Assuming that translation and protein degradation determine constant values for E_i^b and hence that $E_i^b = F_i^b + [\text{dimer}]$, we obtain

$$[\text{dimer}]_{eq} = \frac{1}{2}\left(\frac{k_-}{k_+} + E_1^b + E_2^b - \sqrt{\left(\frac{k_-}{k_+} + E_1^b + E_2^b\right)^2 - 4E_1^bE_2^b}\right). \quad \text{(S2.1)}$$

To calculate the homodimer case, we simply set $E_1^b = E_2^b$. In either case,

$$t_{1/2} = \ln(2)/\left(k_1[\text{dimer}]_{eq}\right) \quad \text{(S2.2)}$$

where k_1 is the rate constant for the reaction catalyzed by the dimer. We use the parameter value $k_- / k_+ = 10^{-3}$ in expression units, so that when stop codon readthrough is 100% and hence E=1 expression unit, dimer formation is realistically stable such that dimers are 31 times as frequent as monomers. Our choice of the parameter value k_1 = 50 per unit concentration, together with our choice of the selective advantage s_2 of [PSI^+], affects the relative fitnesses in our model. As shown in Figure S1, larger values of k_1 mean that optimal expression E_{opt} is lower than complete readthrough E=1. Larger k_1 would therefore be an inappropriate choice, since our model posits readthrough to be unconditionally adaptive in environment 2. Smaller values of k_1 mean that optimal expression levels are unrealistically high. k_1=50 corresponds to optimal expression of the readthrough product a few fold greater than 100% readthrough at existing levels of gene expression. This represents the fact that the adaptive readthrough product is unlikely to

have a fully optimized sequence when first expressed, a defect that can be overcome by moderate overexpression relative to the typical expression level of a gene defined as E=1.
